# Records of Cases Treated in St. Bartholomew's Hospital

**Published:** 1864-06

**Authors:** Frederick C. Skey

**Affiliations:** President of the Royal College of Surgeons, and Surgeon to the Hospital.


ART. III.—*Records of Cases Treated in St. Bartholomew's Hospital.* By FREDERICK C. SKEY, Esq., F. R. S., President of the Royal College of Surgeons, and Surgeon to the Hospital.

In the following paper I propose to take in review some of the more prominent cases that have occupied in my wards in St. Bartholomew's Hospital during the past year. Cases are prominent in a ratio with the interest they raise in the professional mind, and with the amount of instruction obtainable from the study and observation they require. It is the great privilege of the hospital physician or surgeon to revel in multitudes of cases, presenting to his daily increasing experience the almost endless varieties of disease and injury. Endless, indeed, they may be termed; so numerous, so original, so eccentric, that the largest experience can never entire overtake or expound them. But while a large aggregate of cases furnishes the best and indeed the only real source of correct diagnosis, it by no means necessarily infers the treatment to be equally sound. The mental powers which are called into requisition for the purpose of determining the nature of a disease—memory, comparison, &c., are exerted with far less force in the selection of *treatment*. There is but one diagnosis, as there is but one truth; whereas there are varieties in treatment, which spring from early adopted principles, and though engrained in the daily practice of the individual surgeon, may vary largely in the aggregate body of professional men. There are probably no two hospitals in London in which the treatment of the same case would be exactly similar, and in most would differ widely from that adopted in others. Indeed, it is not necessary to carry our investigation beyond the walls of any one institution to observe a diversity of opinion and practice in the province of therapeutics. These opinions cannot all be right, because they are often almost in antagonism. It would be a wholesome condition exacted by the governors of hospitals, that which would require its medical staff to visit similar institutions, under the charge of other hospital officers, to take yearly stock, as it were, of cases, to compare notes, and to extract all available knowledge, whether medicinal or mechanical, adopted in the practice of others. As the case now stands, however, we are all so well content with the correctness of our own views and actions, that I fear the time has scarcely arrived for the adoption of so salutary a practice. The publication of examples of rare or otherwise interesting diseases occurring in the practice of hospitals is an imperative duty of an hospital staff. They belong to the profession, and not to the individual, and he is bound by his allegiance to his profession to give them publicity. Acting in this spirit, I proceed to record such cases of interest, with their treatment, that have come under my observation in St. Bartholomew's Hospital mostly within the year, and on many, perhaps on most, I shall make observations which, failing any better description, may bear that of "clinical."

*Varicose Veins.*

Of cases of varicose veins of the leg every hospital furnishes abundant examples. Whether or not coupled with what is loosely denominated a varicose ulcer, they are often a source of much evil to the subject of them. They are the cause of both weakness and pain. They incapacitate for hard work. But they do not exist alone. Their presence marks a constitution; it is the constitution of debility—a deficiency of power in the acting organs of the circulation. The treatment involves two objects: 1st, the increase of power to these organs; and 2d, the turning the current of the venous circulation into healthier channels. The first is affected by the liberal administration of nutrio-stimulants. The second object has tested the invented faculties of many surgeons. I leave it to others to commend the various schemes adopted by them. I discountenance, from long observation of its incompetency to cure, the employment of the needle, whether through the vein or under it, single or double. It has these objections: 1st, it is not unattended with danger; and 2d, it fails to obliterate the vein, except at the point of its application, mainly because the applications cannot be safely made in numbers proportionate to that of the veins affected. I have at present in St. Bartholomew's Hospital a woman under treatment for varicose veins of the leg, whose limb was jeopardized by the employment of the needle a year ago. A long illness, with severe inflammation and extensive abscesses, followed. The same limb is again under treatment for the original disease. There is no danger in making any number of small eschars on the most projecting surfaces of varicose veins, if made with an escharotic composed of two-fifths of pure potash and three-fifths of powdered lime. This powder, well combined, is made into a paste with alcohol. Whether other escharotics are dangerous in their operation on veins I do not stop to inquire; I only know that the Vienna paste, combined as I have above described it, is not. These eschars may be made in any number proportionate to the extent of the disease. I have treated perhaps 250 cases in the course of the last ten years, and I continue to treat them by the same means. The paste is applied over the most projecting parts of the vein in the in the following manner: through a series of about four layers of adhesive plaster a circle is cut of the size of a threepenny piece or smaller. The influence of the escharotic extends through the vein and it is curious to observe that from the hour of its application the entire vein appears to be obliterated, and is undetectable to the finger on pressure. From ten to twenty-five eschars may be applied between the ankle and the knee. Twenty minutes suffice for the full operation of the escharotic, and an average of one month for the cure. In very weak constitutions the ulcers will heal very slowly, unless well directed efforts be made to give force to the general system.

[TO BE CONTINUED.]

